# The Importance of FDG-PET/CT in Cogan’s Syndrome

**DOI:** 10.4274/mirt.349

**Published:** 2014-06-05

**Authors:** Ebru Örsal, Mahir Uğur, Bedri Seven, Arif Kürşad Ayan, Fatma İçyer, Aslı Yıldız

**Affiliations:** 1 Atatürk University Faculty of Medicine, Department of Nuclear Medicine, Erzurum, Turkey; 2 Atatürk University Faculty of Medicine, Department of Physical Medicine and Rehabilitation, Erzurum, Turkey

**Keywords:** 18FDG, positron-emission tomography/computed tomography, Cogan syndrome, Vasculitis

## Abstract

The present study gives a detailed report of a patient with atypical Cogan’s syndrome with uveitis and sensorineural hearing loss. Cogan’s syndrome is characterized by nonsyphilitic interstitial keratitis and audiovestibular dysfunction. This syndrome can be divided into two groups, typical and atypical, based on the presence of interstitial keratitis. It may sometimes be associated with systemic vasculitis. Fluoro-D-glucose-positron emission tomography/computed tomography (FDG-PET/CT) scanning was used to investigate the presence of vasculitis. With FDG-PET/CT scanning, there is no pathological involvement in the walls of the arteries; thus the patient is protected from aggressive and long term immunosuppressive treatment’s side effects. Hence, we can conclude that FDG-PET/CT may play an important role in excluding the presence of vasculitis.

## Abstract (tr)

Çalışmamızda sensorinöral işitme kaybı ve üveiti olan atipik Cogan sendromlu bir hastayı detaylı bir şekilde ele aldık. Cogan sendromu nonsifilitik interstisyel keratit ve odiovestibuler disfonksiyon ile karakterize bir hastalıktır. Interstisyel keratitin varlığına göre Cogan sendromu tipik ve atipik olmak üzere iki gruba ayrılabilir. Bu sendroma bazen sistemik vaskülit eşlik edebilir. Fluoro-D-glucose-positron emission tomografi/bilgisayarlı tomografi (FDG-PET/BT) taraması sistemik vaskülit varlığının araştırılması amacı ile kullanılabilir. Bu amaçla hastaya yapılan FDG-PET/BT taraması ile arter duvarlarında patolojik bulgu saptanmadı. Böylece hasta uzun süreli ve agresif immünsüpresif tedavinin yan etkilerinden korunmuş oldu. Vaskülit varlığını dışlamada FDG-PET/BT taramasının önemli bir yeri olduğunu söyleyebiliriz.

## INTRODUCTION

Cogan’s syndrome is a rare autoimmune disorder characterized by nonsyphilitic interstitial keratitis and vestibuloauditory system involvement that primarily affects young adults, without a hereditary pattern. The syndrome of nonsyphilitic interstitial keratitis with audiovestibular dysfunction was first reported by Morgan and Baumgartner in 1934 (1,2). Cogan’s syndrome is classified into two groups. The presence of interstitial keratitis indicates typical Cogan’s syndrome and its absence indicates atypical Cogan’s syndrome. The term “atypical Cogan’s syndrome” is used when other types of inflammatory eye disease, including conjunctivitis, uveitis, scleritis, and choroiditis, are associated with the vestibuloauditory abnormalities (3,4). Vollertsen et al. showed that one-third of patients have other affected organs, in the cardiovascular, musculoskeletal, neurological, gastrointestinal or mucocutaneous systems. In addition, systemic vasculitis in any size vessel may be seen in approximately 10% of cases (5,6). Fluoro-D-glucose-positron emission tomography/computed tomography (FDG-PET/CT) has recently been used to diagnose infectious diseases with elevated intracellular glucose metabolism. It has been shown that activated inflammatory cells overexpress glucose transporters and accumulate increased amounts of glucose and structurally related substances such as FDG. Hence FDG-PET/CT is also introduced as a diagnostic way to assess involvement in vasculitis (7,8). In this study we aimed to show findings of FDG-PET/CT in Cogan’s syndrome and emphasize its importance in diagnosing vasculitis.

## CASE REPORT

**Figure 1:**
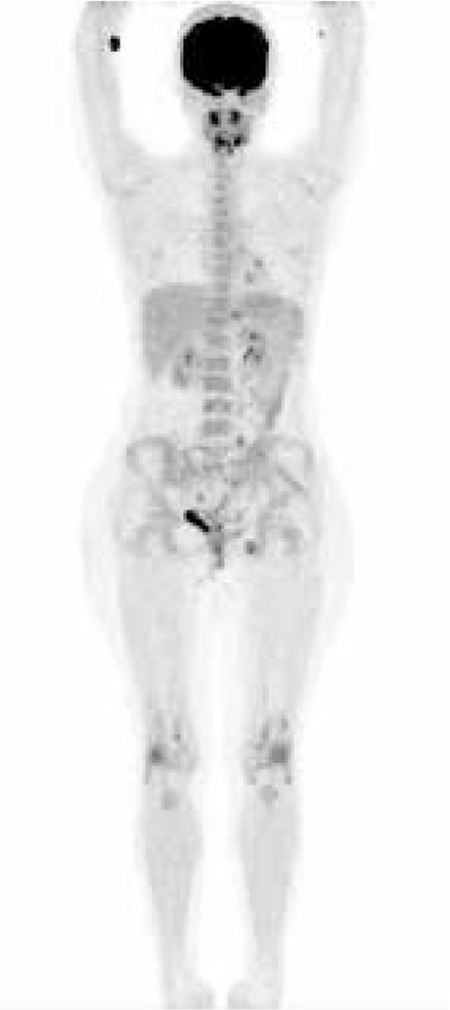
Whole-body maximum-intensity-projection FDG PET image.

A 28-year-old female patient with left knee pain and swelling which occurred two weeks after upper respiratory tract infection was admitted to hospital. The patient had been followed up with uveitis for four years. Nonspecific symptoms like fever, weight loss, fatigue, and arthralgia were present in our patient. Sensorineural hearing loss was detected during follow-up with audiograms. The patient’s laboratory tests revealed the following: erythrocyte sedimentation rate 64 mm/h (normal range 0-20 mm/h), C-reactive protein 51.4 mg/L (normal range 0-5 mg/L), rheumatoid factor 10.6 IU/ml (normal range 0-16 IU/ml). These findings and the presence of both uveitis and sensorineural hearing loss indicated atypical Cogan’s syndrome. Also, arthritis indicated the involvement of other organs. The patient was directed to our center to investigate the presence of vasculitis. PET whole-body imaging was performed 60 minutes after intravenous injection of 10 mCi of FDG. FDG-PET/CT scanning showed no pathological involvement in the walls of the arteries but pathological uptake was found in both knees due to arthritis 18F-FDG PET/CT scanare is shown in Figure 1. 

## LITERATURE REVIEW AND DISCUSSION

Conventional diagnostic approaches such as biopsy and angiography are invasive, and ultrasound and magnetic resonance imaging (MRI) are not adequate for imaging the full extent of vascular involvement in large vessel vasculitis. Metabolic imaging may provide valuable data in the assessment of inflammatory vascular diseases, especially in large vessel arteritides (9). As it has noninvasive and whole body scanning features, FDG-PET/CT investigations may play an important role in the identification of large vessel vasculitis. In Cogan’s syndrome, the presence of vasculitis is vital, because the treatment period and drug doses are planned according to this component. Balink et al. showed that FDG-PET/CT was useful in the evaluation of vasculitis and they found an increased uptake in the aortic arch with FDG-PET/CT. According to the findings obtained by comparing first FDG-PET/CT, the immunosupressive therapy dose was changed (10,11). In this case, we detected the absence of activation in large vessels with FDG-PET/CT. Therefore, low dose steroids and immunosuppressive therapy instead of high dose was planned by clinical physician. After treatment the patient’s complaints decreased. Thus, the patient was protected from aggressive and long term immunosuppressive treatment’s side effects. Consequently, we can say that FDG-PET/CT may play an important role in the diagnosis, therapy design, and evaluation of treatment efficiency in vasculitis. 

## References

[1] Cogan DG (1945). Syndrome of nonsyphilitic interstitial keratitis and vestibulo-auditory symptoms. Arch Ophthalmol.

[2] Gaubitz M, Lubben B, Seidel M, Schotte H, Gramley F, Domscheke W (2001). Cogan’s syndrome: organ-specific autoimmune disease or systemic vasculitis? A report of two cases and review of the literature. Clin Exp Rheumatol.

[3] Haynes BF, Kaiser-Kupfer MI, Mason P, Fauci AS (1980). Cogan’s syndrome: studies on thirteen patients, long-term follow up, and a review of the literature. Medicine (Baltimore).

[4] Grasland A, Pouchot J, Hachulla E, Bletry O, Papo T (2004). Typical and atypical Cogan’s syndrome: 32 cases and review of the literature. Rheumatology (Oxford).

[5] Vollertsen RS, McDonald TJ, Younge BR, Banks PM, Stanson AW, Ilstrup DM (1986). Cogan’s syndrome: 18 cases and a review of the literature. Mayo Clin Proc.

[6] Pagnini I, Zannin ME, Vittadello F, Sari M, Simonini G, Cimaz R, Zulian F (2012). Clinical Features and Outcome of Cogan Syndrome. J Pediatr.

[7] Ishimori T, Saga T, Mamede M, Kobayashi H, Higashi T, Nakamoto Y, Sato N, Konishi J (2002). Increased F18-FDG uptake in a model of inflammation: concanavalin A-mediated lymphocyte activation. J Nucl Med.

[8] Walter MA, Melzer RA, Schindler C, Muller-Brand J, Tyndall A, Nitzsche EU (2005). The value of 18F-FDG PET in the diagnosis of large-vessel vasculitis and the assessment of activity and extent of disease. Eur J Nucl Med Mol Imaging.

[9] Belhocine T, Blockmans D, Hustinx R, Vandevivere J, Mortelmans L (2003). Imaging of large vessel vasculitis with 18FDG PET: illusion or reality? A critical review of the literature data. Eur J Nucl Med Mol Imaging.

[10] Balink H, Bruyn GA (2007). The role of PET/CT in Cogan’s syndrome. Clin Rheumatol.

[11] Allen NB, Cox CC, Cobo M, Kisslo J, Jacobs MR, McCallum RM, Haynes BF (1990). Use of immunosuppressive agents in the treatment of severe ocular and vascular manifestations of Cogan’s Syndrome. Am J Med.

